# Comparative analysis of human respiratory syncytial virus evolutionary patterns during the COVID-19 pandemic and pre-pandemic periods

**DOI:** 10.3389/fmicb.2023.1298026

**Published:** 2023-12-04

**Authors:** Chi-yu Guo, Yu Zhang, Yu-yue Zhang, Wei Zhao, Xiang-lei Peng, Yan-peng Zheng, Yuan-hui Fu, Jie-mei Yu, Jin-sheng He

**Affiliations:** College of Life Sciences and Bioengineering, Beijing Jiaotong University, Beijing, China

**Keywords:** human respiratory syncytial virus, antigenic site, evolutionary pattern, tertiary structure, positive selection

## Abstract

The COVID-19 pandemic has resulted in the implementation of strict mitigation measures that have impacted the transmission dynamics of human respiratory syncytial virus (HRSV). The measures also have the potential to influence the evolutionary patterns of the virus. In this study, we conducted a comprehensive analysis comparing genomic variations and evolving characteristics of its neutralizing antigens, specifically F and G proteins, before and during the COVID-19 pandemic. Our findings showed that both HRSV A and B exhibited an overall chronological evolutionary pattern. For the sequences obtained during the pandemic period (2019–2022), we observed that the HRSV A distributed in A23 genotype, but formed into three subclusters; whereas the HRSV B sequences were relatively concentrated within genotype B6. Additionally, multiple positively selected sites were detected on F and G proteins but none were located at neutralizing antigenic sites of the F protein. Notably, amino acids within antigenic site III, IV, and V of F protein remained strictly conserved, while some substitutions occurred over time on antigenic site Ø, I, II and VIII; substitution S389P on antigenic site I of HRSV B occurred during the pandemic period with nearly 50% frequency. However, further analysis revealed no substitutions have altered the structural conformations of the antigenic sites, the vial antigenicity has not been changed. We inferred that the intensive public health interventions during the COVID-19 pandemic did not affect the evolutionary mode of HRSV.

## Introduction

Human respiratory syncytial virus (HRSV) is a leading cause of acute lower respiratory tract infections in young children and poses a major risk to elderly individuals, imposing a substantial burden on healthcare systems worldwide (Shi et al., 2020; Li et al., 2022). Although HRSV infection can manifest as mild upper respiratory tract illness that typically resolves within 7–10 days without complications, severe lower respiratory tract infections such as bronchiolitis or pneumonia may occur. Infants and individuals with underlying medical conditions or weakened immune systems are particularly vulnerable, which can potentially result in mortality (Falsey and Walsh, 2000; Perk and Ozdil, 2018).

HRSV belongs to the *Orthopneumovirus* genus of the *Paramyxoviridae* family, with a genome consisting of approximately 15,000 nucleotides that encode for 11 viral proteins (Collins et al., 2013). The non-structural proteins NS1 and NS2 play crucial roles in evading the host’s antiviral innate system (Sedeyn et al., 2019), while the attachment glycoprotein (G), a structural protein, acts as a primary target for neutralizing antibodies and plays a critical role in facilitating initial attachment to host cells. Meanwhile, the fusion glycoprotein (F) mediates viral entry by inducing fusion between the viral envelope and cell membranes, and during this process, F undergoes a conformational change from the pre-fusion to post-fusion form (McLellan et al., 2013a,b). The G protein is highly variable and consists of two hypervariable regions flanking a mostly conserved central region. Although HRSV has only one serotype, it can be divided into two subtypes: A and B. Furthermore, based on the second hypervariable region (HVR2) of the G protein, HRSV can be further divided into 13 genotypes, while HRSV B can be classified into 37 genotypes (Muñoz-Escalante et al., 2019, 2021; Kim et al., 2023). The F protein demonstrates conservation across both HRSV subgroups A and B, with multiple major antigenic sites shared between them. Among these sites, site ∅ and V are exclusively present in the pre-fusion form, while site I is only found in the post-fusion form. However, other sites such as site II and IV exist in both forms (McLellan et al., 2013a; Mousa et al., 2017; Bin et al., 2019). Currently, the most prevalent genotypes of HRSV circulating worldwide are ON1 for HRSV A and BA9 for HRSV B (Vieira et al., 2017; Al-Sharif et al., 2020; Song et al., 2023). HRSV tends to be prevalent during winter months lasting 4 or 5 months depending upon different geographical locations, it can also remain sustained throughout the year (Janet et al., 2018). Approximately 33 million cases of acute lower respiratory infections were associated with HRSV, leading to over 100 million deaths annually worldwide among children under 5 years old (Shi et al., 2020; Li et al., 2022).

In late 2019, the COVID-19 pandemic emerged, leading to the implementation of various public health measures. These included wearing facial masks, placing restrictions on public gatherings, and closing borders. Like SARS-CoV-2, HRSV primarily spreads through direct physical contact with infected individuals or via droplets from an infected person. Consequently, the preventive measures had a significant impact on the transmission pattern of HRSV: during the early phase of the COVID-19 pandemic, there was a decline in HRSV-associated hospitalizations due to strict adherence to the preventive measures. However, after their relaxation or lifting in some regions of the world, there has been a resurgence in transmission leading to unexpected peak times for infection (Olsen et al., 2021a; Chow et al., 2023; Ludlow, 2023). For example, in the United States, after lockdown measures were eased, the number of RSV cases was unexpectedly higher during 2022–2023 (Goya et al., 2023). Similarly, in Japan, the Tokyo metropolitan area experienced its highest surge in HRSV cases in 2021 since the establishment of HRSV surveillance in 2003 (Ujiie et al., 2021).

To understand whether changes in HRSV transmission patterns have affected its evolutionary mode, we conducted a comparative evolutionary genomics analysis between pre-pandemic and during-pandemic periods for HRSV circulating globally by using public genomic data sources in this study.

## Materials and methods

### Sequence retrieval and selection

To gather a comprehensive dataset of HRSV sequences, we conducted a search in the NCBI GenBank Database^1^ using “human respiratory syncytial virus” as keywords. Nearly complete HRSV sequences spanning its entire history from 1956 to late December 2022 were downloaded. Important genes such as F, G, and L were truncated from the raw nearly-complete sequences data. In order to ensure high accuracy of the dataset, we excluded sequences that contained low-quality regions with Ns or gaps, as well as those with insertions causing frameshift mutations. Additionally, any sequences that had been manually modified were also omitted from this study.

### Sequence alignment, annotation and distance clustering

The MAFFT 7 online version^2^ was utilized to align all the sequences, followed by manual adjustment of the alignment using MEGA 7 software (version 7.0.26). Additionally, each sequence was annotated with relevant information including the GenBank number, subtype, collection time, and location. Nucleotide pairwise distances between HRSV subtype A and B were calculated by employing the Kimura 2-parameter model in MEGA software. The resulting distance matrix was then subjected to a Multidimensional Scaling Analysis (MDS) algorithm from the R cmdscale package (R version 4.1.2).

### Root-to-tip divergence

TempEst (version 1.5.3, formerly called Path-P-Gen) was implemented to plot the root-to-tip divergence of nucleotide sequences for the G and F genes of HRSV against sampling time, revealing their pattern of divergence and clock-likeness with phylogenetic trees (Rambaut et al., 2016). The trees were constructed by IQTREE software (version 2.1.3) under a general time-reversible (GTR) model that accounted for inter-site rate variation with a discrete gamma distribution. HRSV F and G genes were included to calibrate the molecular clock applied for the analysis. Finally, Prism software was used to process the generated data and create the final graph.

### Phylogenetic analyses

The aligned complete genome sequences of HRSV A and B were separately used for phylogeny analysis. The phylogenetic trees were performed by MEGA 7.0.26 software using maximum likelihood method with Kimura 2-parameter substitution model. Different circulating time and sub-clusters of sequences collected during the COVID-19 period were marked, and proportions of each continent’s contribution to the sub-clusters were calculated.

### SNP calling and selective pressure analysis

Single nucleotide polymorphisms (SNPs) calling was developed by R codes deposited in Github as previously described (Zhang et al., 2022). Statistically supported positively selected sites were localized through Hyphy package. The mixed-effects model of evolution (MEME), a fast unbiased Bayesian approximation (FUBAR), and single-likelihood ancestor counting (SLAC) models were applied for the analysis, and the results from the three models were merged.

### Prediction of tertiary structure for F proteins

Amino acid substitutions on the key antigenic sites of the F proteins were analyzed. Furthermore, the tertiary structures of the proteins with modified amino acids were predicted and aligned to the ones of the prototypes. The online SWISS-MODEL service platform^3^ was utilized to construct the models of the F proteins based on template models derived from RSV F. PYMOL 2.5.1 was used for visualizing and labeling the models. The key antigenic sites of the F protein were plotted on the models.

## Results

### Sequence information

A total of 4,646 nearly complete genome sequences were initially downloaded. After filtering out low-quality and manually modified sequences, there remained 3,886 nearly full-length sequences. Out of these, HRSV A accounted for 2,167 sequences while HRSV B had 1,719. In addition to the full-length sequences, truncation from the raw sequence data resulted in high-quality F and G gene datasets comprising of 4,382 and 4,520 sequences, respectively. Specifically, there were 2,531 and 2,637 high-quality F and G gene sequences, respectively, identified as belonging to HRSV A, and there were a total of 1,851 and 1,883 truncated high-quality F and G genes, respectively, for HRSV B.

To understand the evolutionary characteristics of the virus during the COVID-19 pandemic (2020–2022), we divided sequences dating back to 2008 into 3-year stages. As there were limited sequences available before 2008, we separated them into two periods: from 2000 to 2007 and before 2000. The results revealed that HRSV B had a greater abundance of sequences during the stages of 2014–2016 and 2017–2019, whereas HRSV A dominated in all other time periods with noticeably higher numbers of sequences (Supplementary Figure S1).

In terms of geographical distribution, South America, Oceania, Europe and Africa had approximately equal sequence counts except for Asia with relatively few sequences and North America with large numbers. Specifically, in Africa, the sequence number of HRSV B was slightly higher than that of HRSV A; in Europe, there were roughly equal numbers of HRSV A and B sequences. On all other continents except for North America, where there were over twice as many HRSV A sequences compared to HRSV B (918 versus 435) (Supplementary Figure S1).

### HRSV A and B overall showed chronological specificities, but some sequences in HRSV A were not

**Figure 1:**
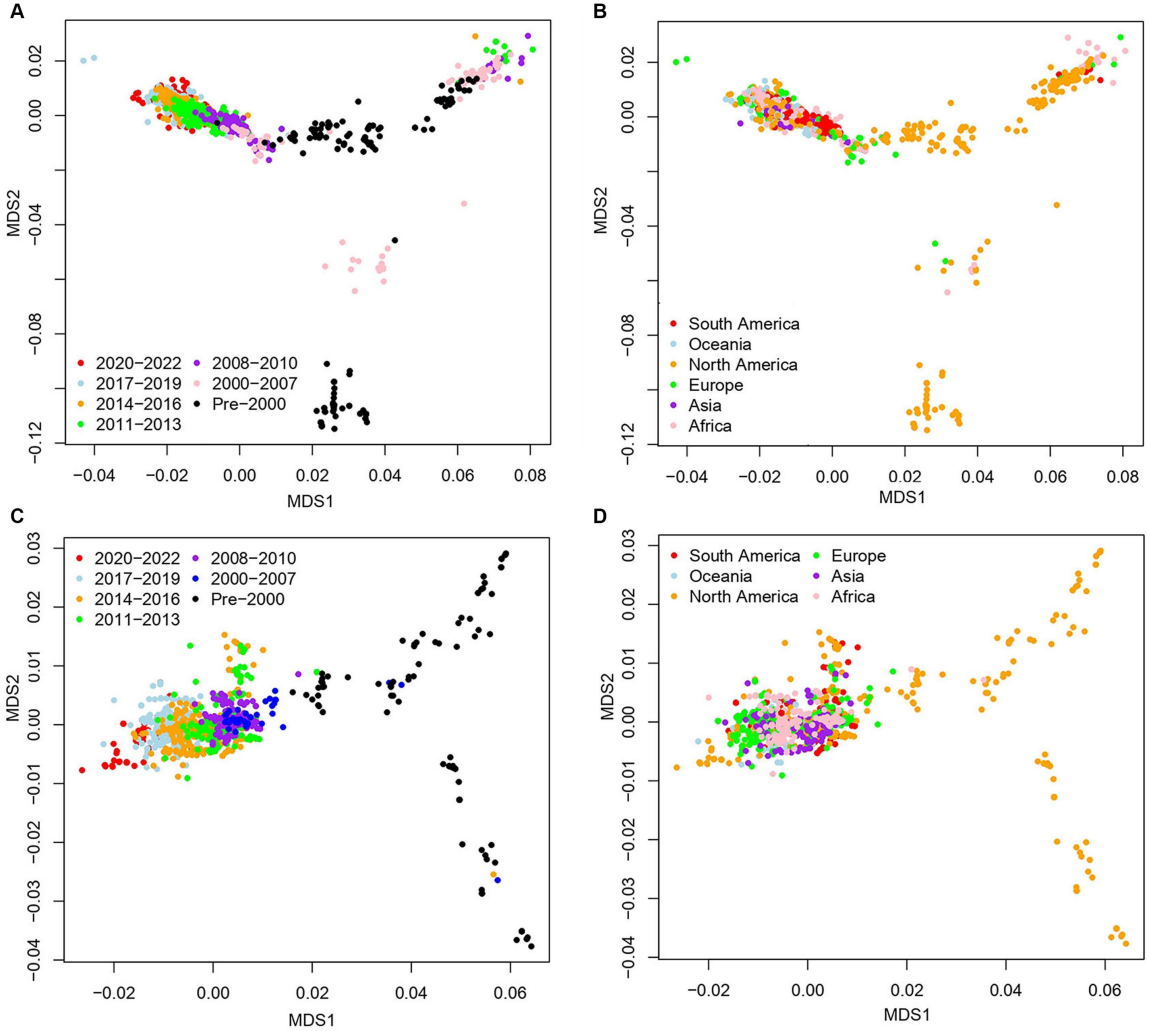
Sequence clustering of G genes for the HRSV A and B by multidimensional scaling (MDS) analysis. **(A,B)** Spatial–temporal distributions of the sequences for HRSV A; **(C,D)** spatial–temporal distributions of the sequences for HRSV B. The sequences overall exhibited time specificity but presented no spatial specificity.

To investigate the genetic characteristics of HRSV A and B, we analyzed their nucleotide distance matrix of the G genes and generated multidimensional scaling (MDS) plots. The results showed that over time, HRSV A underwent evolutionary changes forming a large cluster on the top and two small clusters at the bottom (Figure 1A). The sequences in these two small clusters were primarily associated with North American sequences prior to 2007 (Figure 1B). The big cluster, centered around early pre-2000 sequences, further evolved into two sub-clusters in opposite directions. These sub-clusters did not show any specific regional patterns, although the left sub-cluster demonstrated a chronological evolution. Notably, all HRSV A sequences from the three-year COVID-19 epidemic (2020–2022) were found within the left sub-cluster (Figure 1A). Similarly, HRSV B presented a chronological evolutionary trend where genetic distances between different time periods formed separate clusters (Figure 1C). Sequences before 2000 had more dispersed genetic distances due to prolonged duration and greater diversity during the period. Spatially, these dispersed sequences originated mainly from North America. Furthermore, genetic distances among HRSV B sequences from other continents were closely clustered without clear regional specificity, particularly for those from Africa which had a substantial number of sequences but remained tightly clustered together (Figure 1D).

### G genes had higher evolutionary rates than F, and HRSV A evolving slightly faster than HRSV B

**Figure 2:**
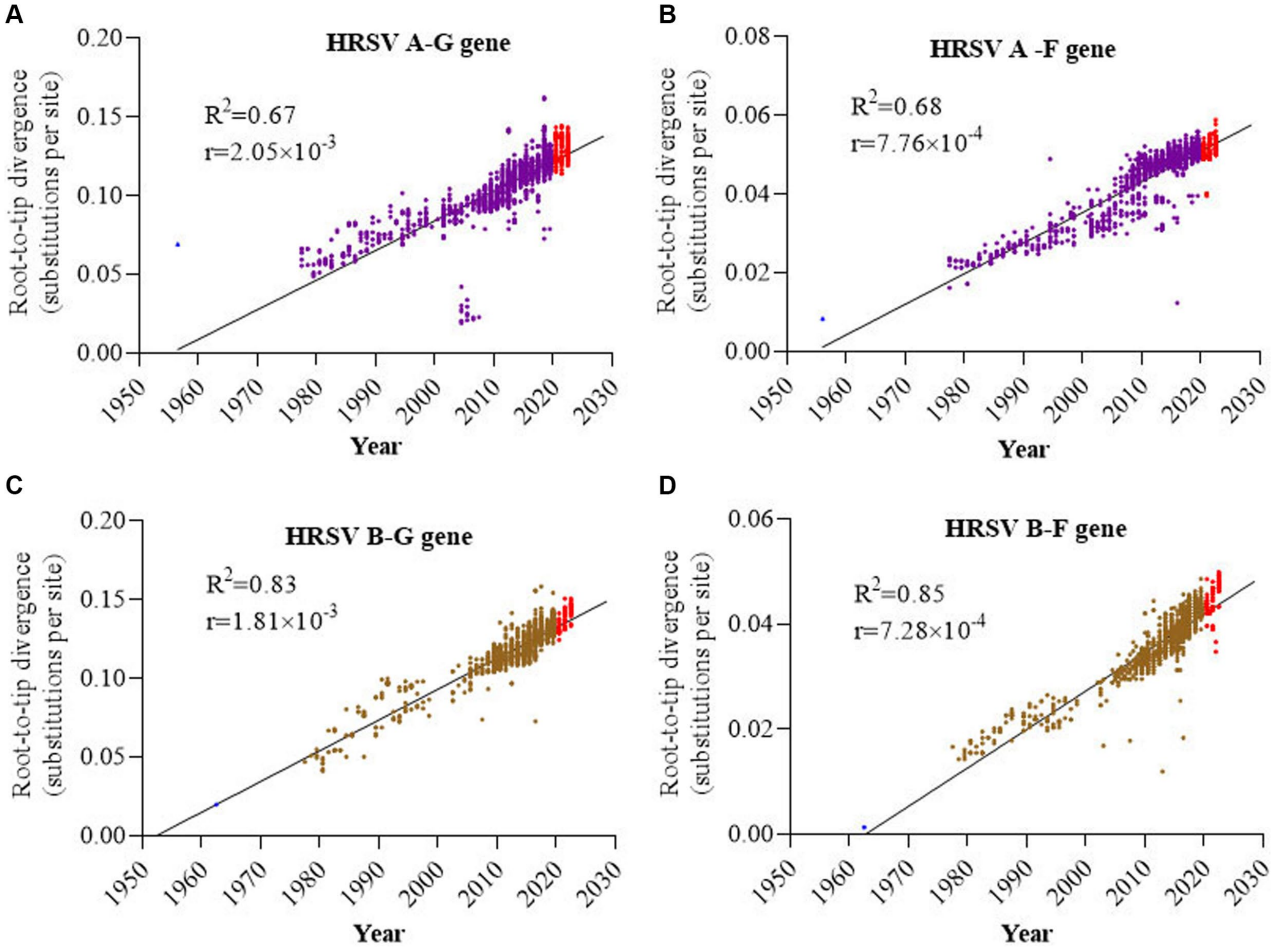
Root-to-tip divergence of the HRSV F and G genes. **(A)** G gene of HRSV A; **(B)** F gene of HRSV A; **(C)** G gene of HRSV B; **(D)** F gene of HRSV B. G genes had higher evolutionary rates than F genes, and HRSV A F and G both had higher evolutionary rates than HRSV B F and G.

Root-to-tip linear regression analyses were performed to examine the genetic divergence of G and F genes in HRSV A and B by utilizing the best-fitting root. The results showed that phylogeny for both G and F sequences of HRSV B exhibited a stronger association (*R*^2^ = 0.83 and 0.85, respectively) compared to those of HRSV A (*R*^2^ = 0.67 and 0.68, respectively), while the association between different subtypes of the same gene (G or F) was similar. All four datasets exhibited positive correlations, indicating their suitability for molecular clock analysis (Figures 2A–D). The evolutionary rates of G genes in both HRSV A and B subtypes were notably higher than those of F genes, with rates of 2.05 × 10^−3^ versus 7.76 × 10^−4^ and 1.81 × 10^−3^ versus 7.28 × 10^−4^ substitutions/site/year, respectively. Furthermore, it was observed that the evolutionary rates of G and F genes was slightly higher in HRSV A than in HRSV B (Figures 2A–D). During the three-year COVID-19 epidemic period, there was an average divergence between sequences of F and G genes when compared to other sequences. Additionally, a small cluster of sequences deviated from others within the G gene sequence in HRSV A, further analysis revealed that these sequences all from the United States spanning years from 2003 to 2006.

### HRSV A and B exhibited chronological evolutionary pattern and sequences during COVID-19 pandemic presented geographical specificity

**Figure 3:**
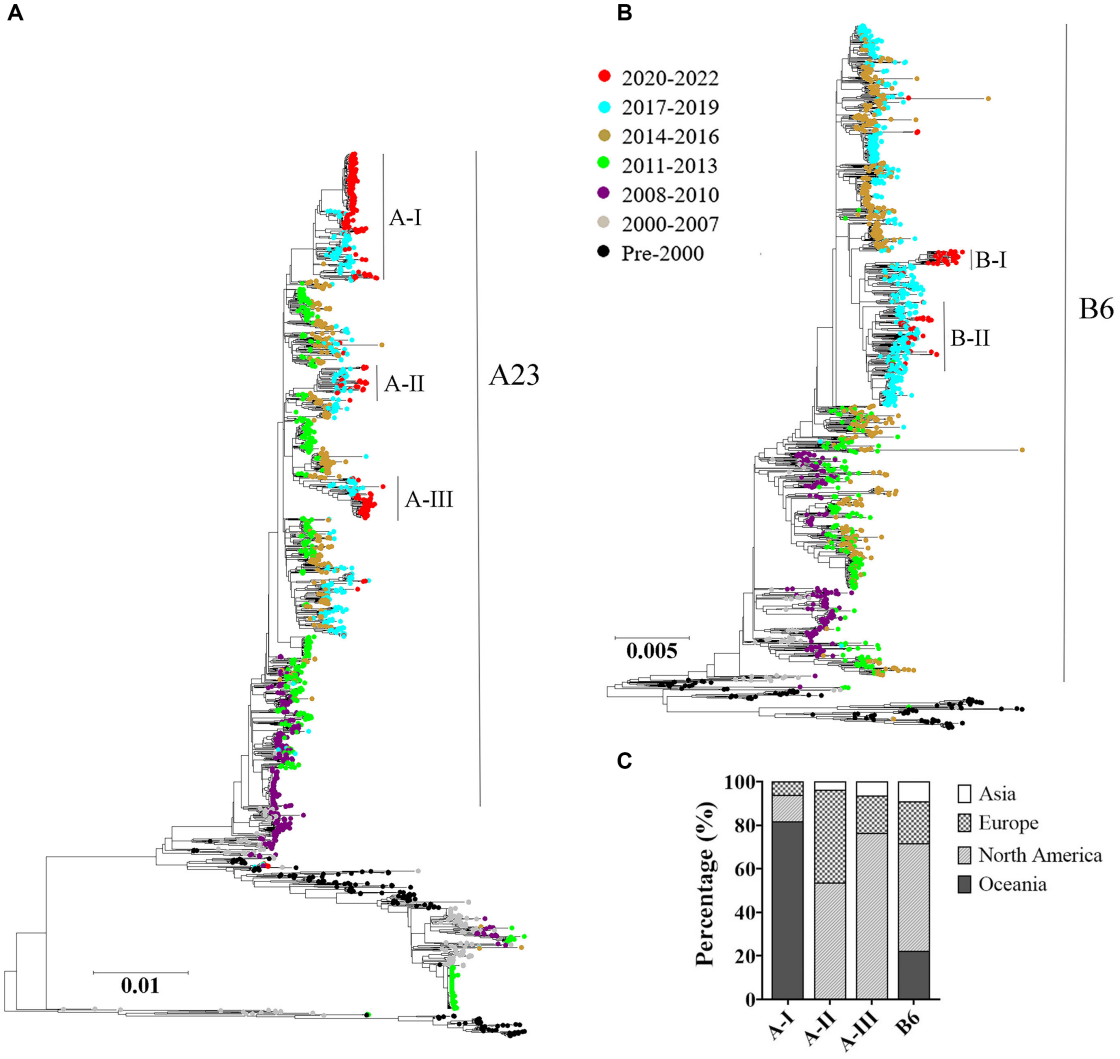
Temporal distributions of evolutionary clusters based on the HRSV complete genome using maximum likelihood method. **(A)** HRSV A phylogenetic tree; **(B)** HRSV B phylogenetic tree; **(C)** geographical distributions of the sub-clusters in COVID-19 pandemic. The genotypes of HRSV A and B overall exhibited a chronological sequential emergence. HRSV A sequences from the pandemic period displayed a geographical specificity.

The maximum likelihood trees were constructed based on the complete genomes of both HRSV serotypes A and B. The results showed a consistent chronological evolutionary trend for both serotypes, where early sequences (pre-2000) clustered at the bottom of the phylogenic trees, while later sequences grouped towards the top. However, there were instances where different subclades co-circulated within the same time period. Notably, from 2011 to 2013 onwards, A23 genotype was predominant for HRSV A, whereas from the year 2000, sequences were mostly B6 genotype for HRSV B. In terms of sequences obtained during 2020–2022, HRSV A and B exhibited a different distribution pattern: the HRSV A formed intro three relatively scattered subclusters; whereas the HRSV B sequences were concentrated (Figures 3A,B). Furthermore, we found that HRSV A sequences during the pandemic period presented a geographical specificity: Oceania-associated sequences distributed in A-I; Asian- and North American-related sequences were found both in A-II and A-III; European sequences distributed predominantly in A-II (Figure 3C).

### The G gene had more SNPs and the G protein had more positively selected sites

**Figure 4:**
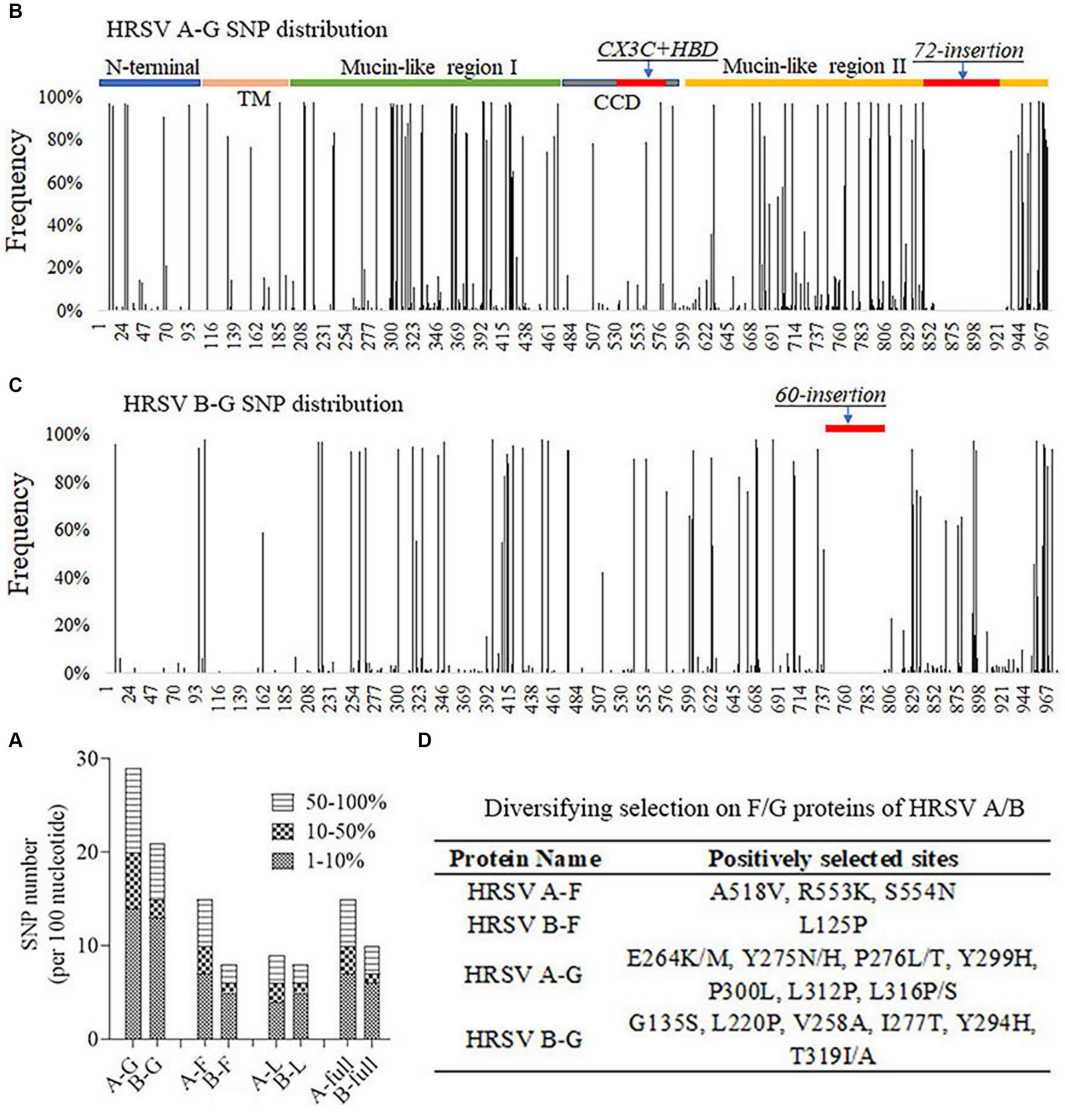
Single nucleotide polymorphism (SNP) distribution and selective pressure analysis. **(A)** The number of SNPs per 100 nucleotides on the viral genomic and important genes. G gene was the most variable. **(B,C)** SNP distributions on the G gene of the HRSV A and B. Mucin-like regions had the greatest number of SNPs and CCD region was relatively conserved. **(D)** Positively selected sites on F and G proteins of HRSV A and B. None sites were located at key neutralizing antigenic sites. TM, Transmembrane region. CCD, Central conserved domain. HBD, Heparin binding domain.

In order to understand the genetic variations of HRSV A and B, SNP callings were performed on their genomes, specifically focusing on the G, F and L genes. An SNP was defined as a site with a mutation frequency greater than 1%. The analysis revealed that HRSV A had more SNPs across the entire genome and in all three genes compared to HRSV B. The majority of observed SNPs occurred at frequencies less than 10%, accounting for approximately 50% of all SNPs; relatively rare were those occurring between 10% to 50% (Figure 4A). On average, HRSV A had 17 SNPs per every 100 nucleotides in its complete genome, while HRSV B had only 12. Although the SNP count per 100 nucleotides for the F genes of both HRSV A and B was equivalent to their full-length sequences, it was lower for their L genes. Notably, the G genes had a considerably higher number of SNPs (Figure 4A), which were distributed throughout the entire sequences with high density in Mucin-like regions I and II, but low density in the central conserved region. Furthermore, an insertion consisting of either a segment of 72-nucleotide or one of 60-nucleotide was found in HRSV A and B, respectively. The occurrence of SNPs in the G gene of HRSV B was found to be less frequent than in HRSV A, particularly at the N-terminus and transmembrane region (Figures 4B,C). Moreover, we further conducted selective pressure analysis on the F and G proteins of both HRSV A and B. Positively selected sites were defined as those supported by all three methods (*p*-value of <0.1 in MEME and SLAC, posterior probabilities of >0.9 in FUBAR). Our results showed a higher number of positive selection sites for the F and G proteins in HRSV A compared to those observed in HRSV B. In particular, the G protein exhibited a markedly greater number of positive selection sites compared to the F protein, with these sites mainly located within the Mucin regions. Notably, none of the amino acids at major antigenic sites of the F protein underwent positive selection. Additionally, there was no simultaneous positive selection observed at the same site for both HRSV A and B regarding their respective F and G proteins (Figure 4D).

### Multiple amino acid substitutions on major antigenic sites of F protein increased over time, but did not alter structural conformation

**Figure 5:**
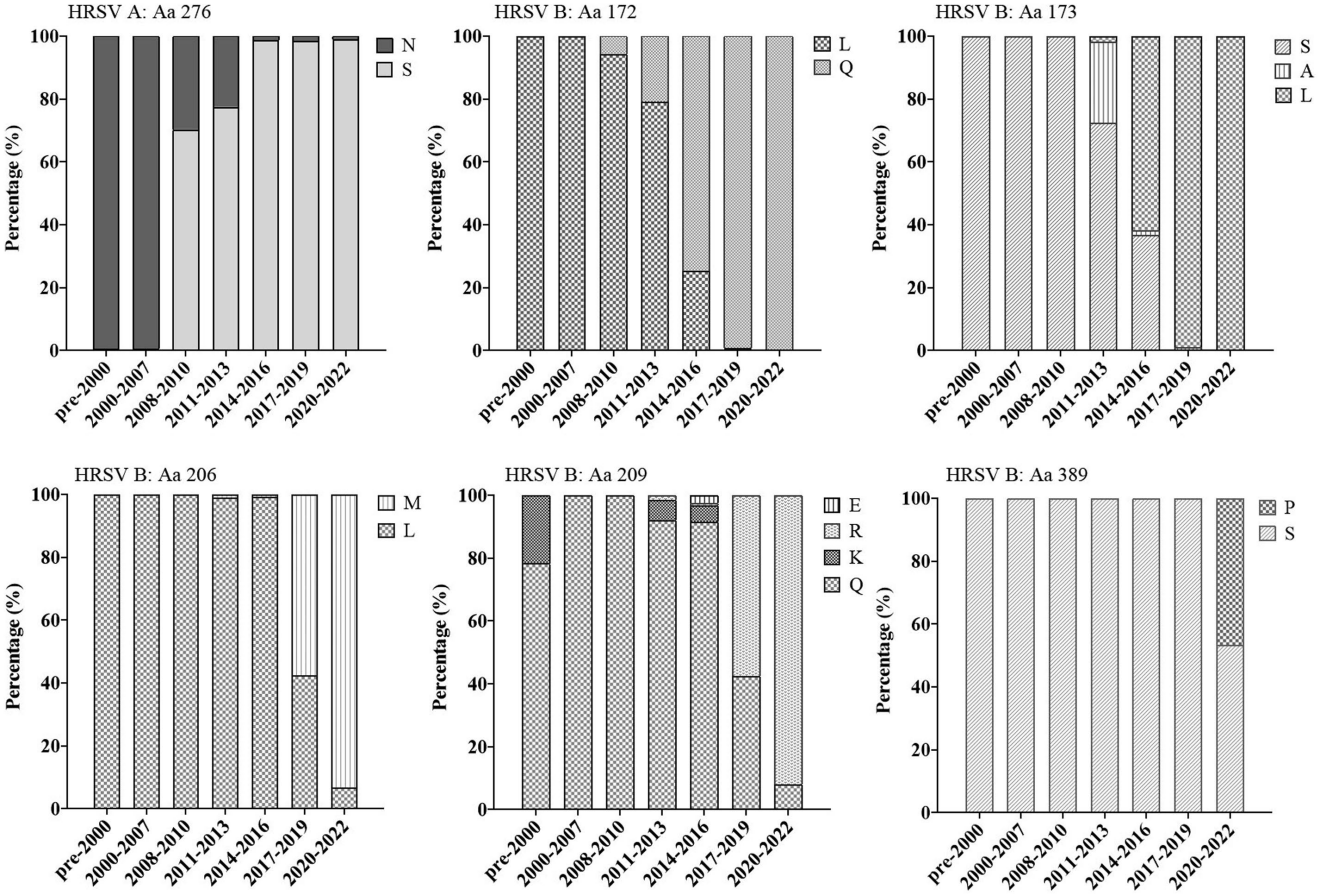
Proportions of substitutions located at the key antigenic sites on F proteins at different time periods. Multiple sites had substitutions and their proportion increased over time. Substitution of S389P occurred during the COVID-19 pandemic period. Aa, amino acid.

The F genes of HRSV A and B had 33 and 37 nonsynonymous mutations, respectively. Among these mutations, three (amino acid (Aa)169, 276 and 384) were situated at the major antigenic sites in HRSV A, while eight (Aa68, 172, 173, 206, 209, 276, 380 and 389) were located at the major antigenic sites in HRSV B. The amino acids on the antigenic sites III, IV and V were strictly conserved, with no changes observed in both HRSV A and B. Out of the 11 identified nonsynonymous mutation sites in this study, five displayed a relatively high frequency of occurrence: four belonged to HRSV B (Aa172, 61.6%; Aa173, 58.6%; Aa206, 23.6%; Aa209, 27.9%), while only one was found in HRSV A (Aa276, 75.7%). On the other hand, the remaining six mutations showed a low frequency of occurrence (less than 5%). Further analysis revealed that amino acid changes at the five high-frequency occurring sites mentioned above, as well as one site with low occurring frequency (i.e., Aa389), exhibited notable temporal features over time. For instance, in HRSV A, Aa276 was exclusively composed of N before time period of 2008–2010, and starting from 2014 to 2016, it underwent a significant replacement with S almost entirely replacing N. While in HRSV B, S was predominated in this position (Aa276), with only a small percentage being N (at 1.73% occurring frequency). Additionally, Aa172 and Aa173 in HRSV A were strictly conserved as L and S, respectively. However, in HRSV B, these two sites experienced substitutions to Q and to L, respectively. By the period of 2017–2019, they had undergone complete changes. Similarly, residue I at Aa206 and residue K at Aa209 were strictly conserved in HRSV A. In contrast, they were L and Q, respectively, in HRSV B but simultaneously changed to M and R since the period of 2017–2019. Concerning the residue at position Aa389, it was P for HRSV A and S for HRSV B with both residues being well-conserved. However, during the COVID-19 epidemic period, a large proportion of sequences in HRSV B changed from S to P (Figure 5).

**Figure 6:**
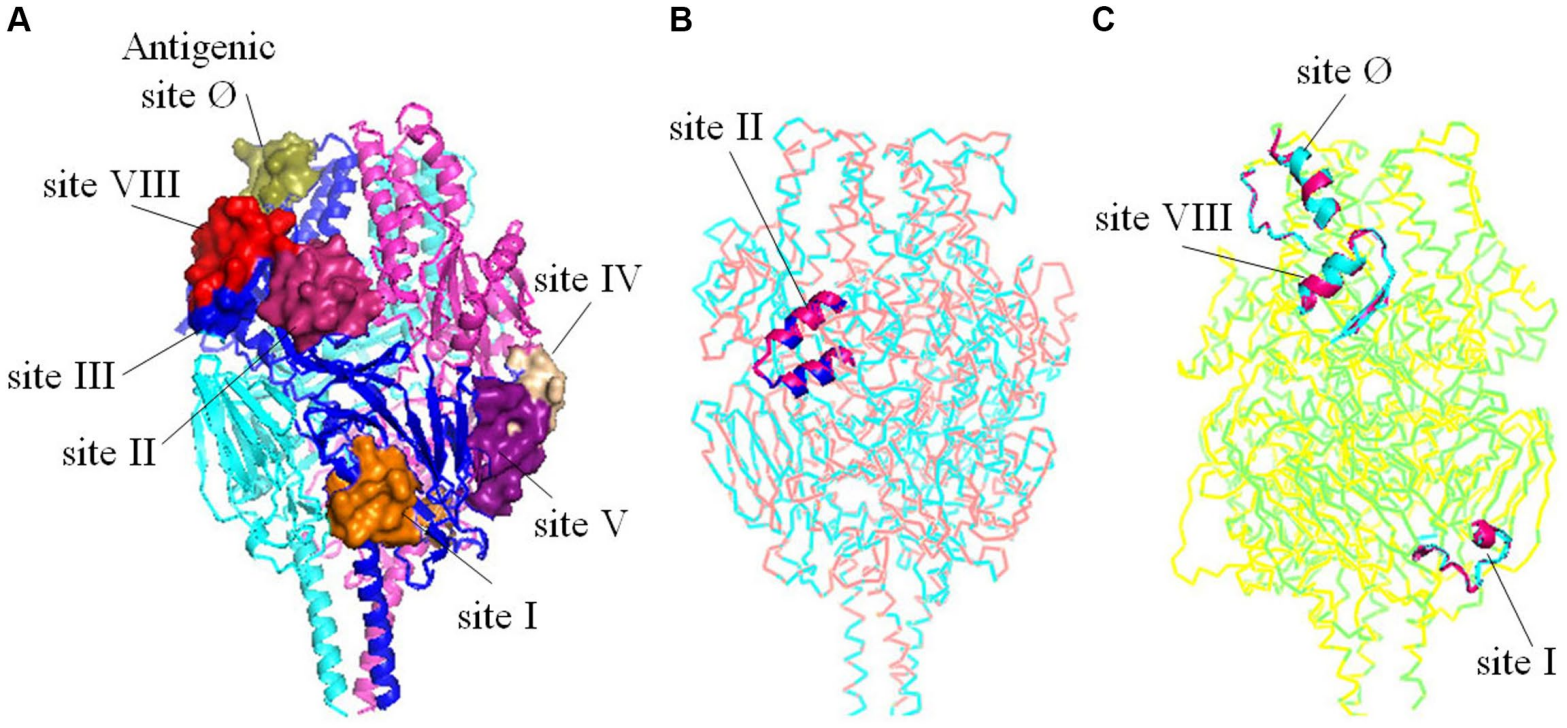
Tertiary structures comparisons of the trimeric F proteins. **(A)** Three-dimensional structure diagrams of the major antigenic sites (Ø-V, VIII) on the prototype of F protein. **(B)** Structural alignment between the mutant with substitutions on the antigenic site II and the prototype of HRSV A. **(C)** Structural alignment between the mutant with substitutions on the antigenic site Ø, I, VIII and the prototype of HRSV B. The substitutions did not alter the tertiary structures of the key antigenic sites.

To investigate the potential impact of amino acid substitutions at major antigenic sites in F proteins of HRSV A and B on their spatial configuration and subsequent antigenic properties, tertiary structures of the F proteins were generated and compared. Our findings revealed that despite substitutions at Aa276 (N to S) in antigenic site II in HRSV A, as well as Aa172 (L to Q) and Aa173 (S to L) in antigenic site VIII, Aa206 (I to M) and Aa209 (Q to R) in antigenic site Ø, and Aa389 in antigenic site I in HRSV B, no changes were observed in the three-dimensional structures of the F proteins (Figure 6). Furthermore, other neutralizing antigenic sites (III, IV, and V) exhibited high conservation across both HRSV A and B, with no discernible amino acid substitutions.

## Discussion

The COVID-19 outbreak has resulted in notable modifications to the incidence pattern of HRSV, impacting both viral prevalence and seasonal trends (Olsen et al., 2021b). Following the lifting of local COVID-19 restrictions, there was a notable surge in HRSV activity, particularly among young children (Falsey et al., 2022; Riepl et al., 2023). This study aimed to delineate the similarities and differences in evolutionary patterns of HRSV genomes, with a specific focus on the F and G genes during pre-pandemic and pandemic periods.

It has been documented that the circulation patterns of HRSV encompass continuous seasons where HRSV A prevails, as well as alternating periods with a predominance of either HRSV A and B (Cantu-Flores et al., 2022). Furthermore, the correlations between HRSV and weather conditions have exhibited variations across different geographic locations (Haynes et al., 2013). In this study, we observed disparities in the proportions of HRSV A and B during distinct time periods. Specifically, sequences belonging to HRSV B were more prevalent than those attributed to HRSV A in the 2014–2016 and 2017–2019 periods. Conversely, in other time intervals, including the COVID-19 pandemic, there was a higher prevalence of HRSV A compared to HRSV B. However, it is important to consider possible sampling bias due to limitations in sample size and geographical coverage.

This study revealed substantial genetic divergence in the G genes of HRSV A and B, especially evident in early sequences predating 2000. Post-2000 sequences exhibited a clear chronological evolution pattern for HRSV B, while HRSV A diverged into two distinct subsets. One subset exhibited temporal order, while the other indicated the simultaneous presence of multiple subclusters. This finding was consistent with a previous study demonstrating that different lineages of ON1 genotype co-circulate without apparent temporal or geographical distribution tendencies (Song et al., 2023). Geographic analysis demonstrated that North America had the highest diversity in terms of sequence variations due to its larger sample size compared to other regions.

Previous studies have shown that the discriminatory power of partial or complete sequences from the highly variable G gene was limited in characterizing HRSV transmission, it has been suggested to use whole genome data in defining HRSV genotypes (Agoti et al., 2015; Ramaekers et al., 2020; Chen et al., 2022). Therefore, in this study, we employed full-genome sequences for phylogenetic analysis, and used the newly established criteria for defining genotypes within HRSV-A and B (Ramaekers et al., 2020). The result revealed a chronological sequential emergence of both HRSV A and B genotypes throughout history. During the COVID-19 pandemic period, HRSV A sequences scattered in three different genotypes and exhibited geographical specificity, while HRSV B sequences all centered in one genotype. This observation is not surprising since previous studies have revealed an apparent pattern of geographical clustering among HRSVs (Robertson et al., 2021). The geographical-specific distribution of HRSV may be associated with different climatic conditions in various areas, as previous studies have indicated that climate may play a crucial role in driving viral epidemics (Pitzer et al., 2015; Baker et al., 2019).

Studies have estimated that the evolutionary rate of HRSV B was higher than that of HRSV A, with the G gene evolving at a faster pace compared to the F gene (Bose et al., 2015; Chi et al., 2019; Yu et al., 2021). During the COVID-19 pandemic period, it has been reported that both HRSV A and B continue to evolve, but with a faster evolutionary rate observed in HRSV B compared to HRSV A (Yan et al., 2023). However, our study found that the mean evolutionary rates for both G and F genes were slightly faster in HRSVA compared to those in HRSV B. Additionally, we identified an approximately 2.5 times faster evolutionary rate for the G gene when compared to the F gene (Chi et al., 2019). The discrepancy between our findings and previous studies regarding evolutionary rates may be attributed either to larger sample sizes or biases introduced through different calculation models used. Furthermore, we observed certain deviating sequences from other regions and time periods with HRSV A sequences from the United States between 2003 and 2006. However, these particular sequences were not detected in any other regions or subsequent time periods. We hypothesized that this observation could be due to founder effects. Interestingly, sequence divergence during the COVID-19 pandemic period was comparable to those from other time periods, indicating preventive measures such as mask-wearing and social distancing did not introduce additional pressures on the evolution of HRSV.

RNA viruses are known to have a higher tendency for mutation compared to DNA viruses due to the lack of proofreading activity in their RNA-dependent RNA polymerases (Peck and Lauring, 2018). In particular, the G gene of HRSV is considered the most variable region, and it contains two highly variable mucin-like domains flanking the central region, making it a valuable characteristic for studying viral evolution (McLellan et al., 2013b). Our study found a remarkably higher mutation rate in the G genes of both HRSV A and B. Specifically, we found that there were 2–3 times more SNPs occurring per 100 nucleotides in the G genes compared to the full genome length, as well as the F and L genes. Moreover, these SNPs were mainly located within the mucin-like domains of the G genes. Further selective pressure analysis suggested that positive selection acting on proteins (F and G) containing neutralizing antigenic sites in both HRSV A and B. It is worth noting that different studies may employ various models for RSV evolution when conducting selective pressures analysis, resulting in slightly different outcomes (Pretorius et al., 2013; Tan et al., 2013). Our study took a conservative approach by defining positive selection sites only when they were detected by all three models (MEME, FUBAR and SLAC). The presence of positively selected sites at specific codon positions suggested that HRSV has been evolving over time through selective processes favoring new variants.

The F protein of HRSV is highly conserved and plays a crucial role in membrane fusion and infection (McLellan et al., 2013a). It exists in two primary conformations: a pre-fusion form (preF), which is metastable, and a post-fusion form (post F), which is stable (Crank et al., 2019). The F protein contains seven major neutralizing antigenic sites (Ø-V, VIII) with sites Ø, V, and VIII exclusively present on the pre-F. Site III is predominantly located in the preF, while the remaining sites are found in both preF and postF forms (Graham, 2017; Jones et al., 2019). Neutralizing antibodies targeting the F protein are critical for developing RSV vaccines aiming to prevent infection (Ruckwardt et al., 2019). Recent neutralization assays conducted in China revealed that pediatric patients infected with different subtypes/genotypes of HRSV exhibited varying antibody titers against different subtypes/genotypes of HRSV (Zhou et al., 2023). Our study identified amino acids changes at antigenic sites Ø, I, II and VIII within the F protein. These alterations have potential implications for viral antigenicity. Therefore, we conducted further predictions and annotation of tertiary structures for F proteins to clarify the impact of these amino acid changes at important sites on major antigenic sites. The results showed that none of the observed substitutions led to changes in spatial structure within the identified antigenic sites, indicating there were no alterations in the antigenic properties of the F protein due to these specific amino acid changes. It was speculated that those substitutions may not be driven by host immune pressure but rather related more to viral fitness.

In conclusion, this study investigated the evolving characteristics of HRSVs during pre-pandemic and COVID-19 pandemic periods. Our findings revealed that although there were certain differences in genetic variation and prevalence features between HRSV A and B, their evolutionary patterns remained similar before and during the pandemic. Notably, amino acid substitutions at nuetralizing antigenic sites of F protein exhibited temporal specificity. However, these substitutions did not result in any discernible alterations in viral biological properties but may have been associated with viral adaptability. It is speculated that the stringent mitigation measures implemented during the pandemic effectively controlled COVID-19 incidence and excess mortality without placing additional immune pressure on HRSV. Nonetheless, continuous monitoring of genomic variations within HRSV remains crucial to generate scientific data for designing effective vaccines.

## Data availability statement

The datasets presented in this study can be found in online repositories. The names of the repository/repositories and accession number(s) can be found in the article/Supplementary material.

## Author contributions

C-yG: Data curation, Formal analysis, Methodology, Writing – original draft. YZ: Data curation, Formal analysis, Methodology, Software, Writing – original draft. Y-yZ: Data curation, Formal analysis, Investigation, Methodology, Writing – original draft. WZ: Data curation, Formal analysis, Methodology, Validation, Writing – original draft. X-lP: Data curation, Formal analysis, Resources, Software, Writing – review & editing. Y-pZ: Data curation, Formal analysis, Methodology, Writing – review & editing. Y-hF: Data curation, Funding acquisition, Investigation, Software, Writing – review & editing. J-mY: Conceptualization, Project administration, Supervision, Visualization, Writing – review & editing, Writing – original draft. J-sH: Conceptualization, Funding acquisition, Project administration, Validation, Visualization, Writing – review & editing.

## Footnotes

^1^http://www.ncbi.nlm.nih.gov/genbank

^2^https://mafft.cbrc.jp/alignment/software/

^3^https://swissmodel.expasy.org/
