# Supplementary figures and images for: Comparative analysis of human respiratory syncytial virus evolutionary patterns during the COVID-19 pandemic and pre-pandemic periods
Source: Front Microbiol. 2023 Dec 4;14:1298026. doi: 10.3389/fmicb.2023.1298026 (PMC10725919; doi:10.3389/fmicb.2023.1298026)

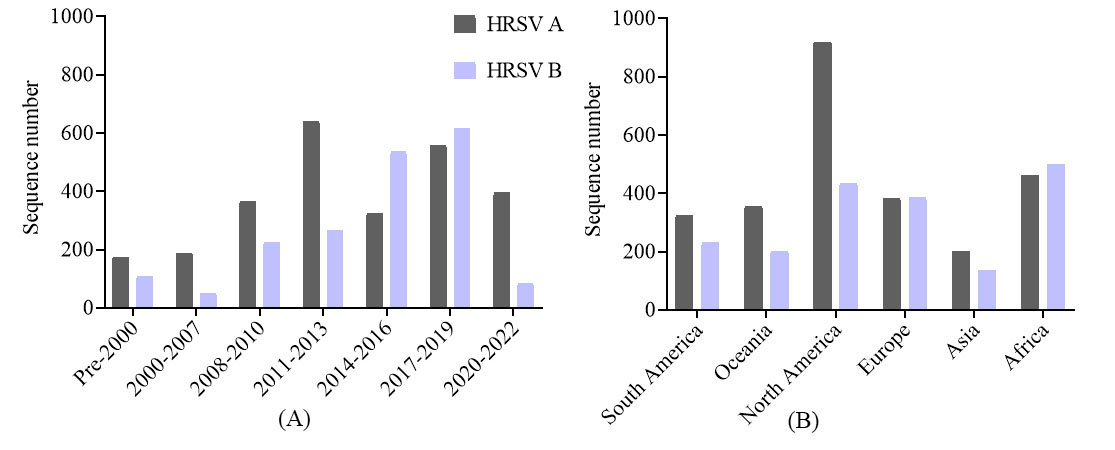

Supplement: Supplementary file 3 [file Image_1.JPEG]
